# Cholinergic Receptor Modulation as a Target for Preventing Dementia in Parkinson’s Disease

**DOI:** 10.3389/fnins.2021.665820

**Published:** 2021-09-20

**Authors:** Alexandre Iarkov, Cristhian Mendoza, Valentina Echeverria

**Affiliations:** ^1^Laboratorio de Neurobiología, Facultad de Ciencias de la Salud, Universidad San Sebastián, Concepción, Chile; ^2^Research & Development Service, Bay Pines VA Healthcare System, Bay Pines, FL, United States

**Keywords:** cotinine, dementia, Parkinson’s disease, cholinergic interneurons, medium spiny neurons, striatum

## Abstract

Parkinson’s disease (PD) is a neurodegenerative condition characterized by the loss of dopaminergic neurons in the substantia nigra pars compacta (SNpc) in the midbrain resulting in progressive impairment in cognitive and motor abilities. The physiological and molecular mechanisms triggering dopaminergic neuronal loss are not entirely defined. PD occurrence is associated with various genetic and environmental factors causing inflammation and mitochondrial dysfunction in the brain, leading to oxidative stress, proteinopathy, and reduced viability of dopaminergic neurons. Oxidative stress affects the conformation and function of ions, proteins, and lipids, provoking mitochondrial DNA (mtDNA) mutation and dysfunction. The disruption of protein homeostasis induces the aggregation of alpha-synuclein (α-SYN) and parkin and a deficit in proteasome degradation. Also, oxidative stress affects dopamine release by activating ATP-sensitive potassium channels. The cholinergic system is essential in modulating the striatal cells regulating cognitive and motor functions. Several muscarinic acetylcholine receptors (mAChR) and nicotinic acetylcholine receptors (nAChRs) are expressed in the striatum. The nAChRs signaling reduces neuroinflammation and facilitates neuronal survival, neurotransmitter release, and synaptic plasticity. Since there is a deficit in the nAChRs in PD, inhibiting nAChRs loss in the striatum may help prevent dopaminergic neurons loss in the striatum and its pathological consequences. The nAChRs can also stimulate other brain cells supporting cognitive and motor functions. This review discusses the cholinergic system as a therapeutic target of cotinine to prevent cognitive symptoms and transition to dementia in PD.

## Introduction

Parkinson’s disease (PD) is a severe neurodegenerative condition characterized by the death of dopaminergic motor and non-motor symptoms leading to locomotor impairment, loss of cognitive function, dementia, psychiatric disorders, and premature death (Gelb et al., 1999; Leverenz et al., 2009; Subramaniam and Chesselet, 2013; Grover et al., 2015; Aarsland et al., 2017; Martin-Jimenez et al., 2017; Poewe et al., 2017; Walker et al., 2019; Wichmann, 2019; Hussein et al., 2021).

More than a century of studies in PD achieved breakthrough discoveries in the etiology of this disease and the role of cholinergic neurons (Quik et al., 2015b; Jurado-Coronel et al., 2016; Rizzi and Tan, 2017; Tanimura et al., 2018; Ztaou and Amalric, 2019; Iarkov et al., 2020; Liu, 2020). These studies indicated that the etiology of PD is not entirely clear and has a complex and multifactorial nature (Olanow and Tatton, 1999; Takahashi and Yamada, 1999; Thomas and Beal, 2007; Guttuso et al., 2019b; O’Callaghan and Miller, 2019; Hasan et al., 2020). Etiological risk factors are considered a combination of age, gender, genetic background, and environmental factors (Carvey et al., 2003; Thomas and Beal, 2007; Pavlou and Outeiro, 2017; Videira and Castro-Caldas, 2018; Guo et al., 2019; Mehra et al., 2019; Delic et al., 2020; Kline et al., 2020). Nevertheless, less than 15% of PD cases have a family history, and most of them are sporadic and seemingly caused by deleterious environmental factors acting synergically with susceptibility genes to affect the striatum activity (Foltynie et al., 2002a, b; Ferrer et al., 2011; Wirdefeldt et al., 2011; Deng et al., 2018; Guo et al., 2019). However, much remains unclear, and effective treatments have yet to be developed based on innovative new strategies (Liu et al., 2010; Campos et al., 2011; Schapira, 2011; Irwin et al., 2016; Wang et al., 2017; Shimohama and Kawamata, 2018; Iarkov et al., 2020). A decline of the flow of information from midbrain dopaminergic neurons to the striatum, limbic, and cortical regions and a deficiency of dopamine (DA) in these structures are central events triggering PD (Alexander, 2004; Cerasa et al., 2016; Anderkova et al., 2017; Galantucci et al., 2017). The decrease of incoming dopaminergic input disrupts complex regulatory mechanisms in the overlying structures (Gale et al., 2008; Schapira and Jenner, 2011; Singh et al., 2017). Deficiency of DA can arise due to the neuronal death or synaptic dysfunction of dopaminergic neurons in the midbrain (Schulz-Schaeffer, 2010, 2015; George et al., 2013). The striatum contains mainly GABAergic medium spiny neurons (MSN) and large aspiny choline interneurons ChIs (Alexander et al., 1986; Conti et al., 2018; Tanimura et al., 2018; Martel and Apicella, 2021). At the cellular level, DA deficiency induces an imbalance of the activity of different MSN populations resulting in motor and behavioral disturbances (Alexander, 2004; Taverna et al., 2008; Tozzi et al., 2011; Gallo, 2019; Iarkov et al., 2020). MSNs expressing the DA receptor 2 (D2R) will decrease their activity, while neurons expressing the DA receptor 1 (D1R) will increase it (Taverna et al., 2008; Tozzi et al., 2011; Gallo, 2019). Alteration of the indirect and direct pathways to globus pallidus internal (GPi)/Substantia nigra pars reticulata (SNpr) impairs the communication of the thalamus with the motor cortex resulting in motor dysfunction (Alexander et al., 1986; Bjorklund and Dunnett, 2007; Rodriguez-Sabate et al., 2021). In other words, an enhanced excitatory output from the subthalamic nucleus (STN) increases the activity of the GP that induces an anomalous inhibitory outflow to the thalamus and brain stem areas (Braak and Braak, 2000; Cerasa et al., 2016; Iarkov et al., 2020; van Nuland et al., 2020). The inhibition of the thalamus affects the thalamocortical communication triggering movement abnormalities characteristic of PD, such as bradykinesia (DeLong, 2000; Poewe et al., 2017; Wichmann, 2018; Reich and Savitt, 2019).

Though PD is a progressive neurodegenerative disease that is mainly considered a clinically dominant movement disorder, it also has noticeable non-motor symptoms such as psychiatric signs of depression, anxiety, and cognitive impairment, some of which may appear even before the motor ones (Hemmerle et al., 2012; Lindqvist et al., 2012; Lohle et al., 2019; Mendonca et al., 2020; Carey et al., 2021; Hussein et al., 2021; Kwon et al., 2021).

Many research groups have investigated the prevalence, sex differences, morphological and functional changes, and biomarkers to predict the progression from cognitive impairment to dementia in PD (Braak et al., 2005; Anang et al., 2017; Hoogland et al., 2017; Ye et al., 2017; Cholerton et al., 2018; Friedman, 2018; Hussain and Camicioli, 2018; Lanskey et al., 2018; Renouf et al., 2018; Zou et al., 2018; Agelink van Rentergem et al., 2019; Berman and Miller-Patterson, 2019; Chondrogiorgi et al., 2019; Chung et al., 2019; Palermo et al., 2019a, b; Yoo et al., 2019; Byeon, 2020).

Almost two decades ago, Braak et al. (2005) found an association between cognitive status and the neuropathologic stages of PD in patients with the sporadic form of the disease. The authors assessed Lewy bodies (LBs) immunoreactive for α-SYN and neuropathological markers for comorbidities such as Alzheimer’s disease (AD) that could be contributing to cognitive decline. The authors divided the patients into groups from marginally impaired cognition to severe dementia according to the Mini-Mental State Examination (MMSE) scores. The results showed that MMSE scores positively and linearly correlated with ascending neuropathologic stages (Braak et al., 2005). Cognitively impaired patients showed higher levels of AD-like neuropathology, including beta-amyloid (Aβ) deposition than cognitively intact patients. MMSE scores did not correlate significantly with disease duration, age at disease onset, or death. The authors concluded that a decrease in MMSE scores between the disease stages 3 to 6 raises the risk of developing dementia during PD progression (Ross et al., 1996; Braak et al., 2005). However, in some patients, cognitive decline develops in the absence of substantial PD-related cortical pathology and, on the contrary, in other patients, extensive cortical neuropathology does not unavoidably lead to cognitive decline and dementia (Green et al., 2002; Braak et al., 2005; Leverenz et al., 2009). Further studies have given more insight into the mechanisms and morphological correlations of cognitive impairment progression to dementia (Aybek et al., 2009; Foster et al., 2013; Aarsland et al., 2017; Lanskey et al., 2018).

On the other hand, other non-motor symptoms, including anxiety and depression, and impulse control disorder, and psychosis, affect many patients, with a prevalence of 50–80%, often appearing at the early stages of the disease is only partially treated by conventional treatments such as L-DOPA and new treatments have been tested (Bonito-Oliva et al., 2014; Titova and Chaudhuri, 2018; Eisinger et al., 2019; Hussein et al., 2021). Experimentally, non-motor symptoms can be induced in mice by bilateral injection of the toxin 6-hydroxydopamine (6-OHDA) in the dorsal striatum. This mouse model of PD-like pathology shows only slight gait modifications, with no horizontal motor activity changes as tested in the open-field test. However, The treated mice showed depressive-like behavior such as increased immobility in the forced swim and tail suspension tests.

Additionally, mice showed anxiety, expressed as a reduced time spent in the open arms in the classic anxiety test elevated plus maze test and increased thigmotaxis in the open-field test. L-DOPA did not decrease depressive- and anxiety-like behaviors. Reboxetine, a noradrenaline reuptake inhibitor, reverted the depressive and anxiogenic effects. However, desipramine used to preserve noradrenaline neurons, when administered before injection of 6-OHDA, did not modify the resultant depressive- and anxiety behaviors. The authors concluded that mood-related disorders were not due to a decrease in noradrenaline (Bonito-Oliva et al., 2014). Last decade studies have indicated the involvement of alteration of the serotoninergic system and its components, such as the serotonin receptors, with the appearance of depression in PD (Ballanger et al., 2012; Bonito-Oliva et al., 2014; Maillet et al., 2016). One of these studies used positron emission tomography (PET) and (18)F (Roselli et al., 2010) MPPF, a selective serotonin 1A receptor antagonist, to investigate whether changes in this receptor activity at the postsynaptic site were involved in the pathophysiology of depression. Compared with non-depressed parkinsonian patients, depressed patients showed a lower tracer uptake in the left hippocampus, the right insula, the left superior temporal cortex, and the orbitofrontal cortex. Compared with controls, non-depressed parkinsonian patients presented a reduced F-18 MPPF uptake bilaterally in the frontal cortex and the right ventral striatum and insula. Compared with controls, F-18 MPPF uptake was decreased in depressed parkinsonian patients in the left dorsal anterior cingulate and orbitofrontal cortices, in the right hippocampal region, and the temporal cortex. The imaging data suggest that serotonin 1A receptor dysfunction in the limbic system may underly depression in patients with PD (Ballanger et al., 2012; Bonito-Oliva et al., 2014; Maillet et al., 2016). The mechanism of action of various neuroprotective strategies to prevent PD is under investigation; however, efficacious new therapeutic approaches still need to be discovered (Guo et al., 2019; Jurado-Coronel et al., 2019; Iarkov et al., 2020).

## The Role of Lewy Bodies in PD

The progressive appearance of protein deposits called Lewy bodies often accompanies the loss of dopaminergic neurons in various brain regions (Schulz-Schaeffer, 2010; Mehra et al., 2019). These deposits contain elevated misfolded α-synuclein (α-SYN) oligomers and aggregates, neurofilaments, and ubiquitin inside neurons and glia (Braak et al., 1998; Martin et al., 2012). Although the role of Lewy bodies in the development of PD is still unknown, the neuropathological diagnosis of PD was base on its detection and quantification (Beach et al., 2008, 2009). Intriguingly, not always neurodegeneration of dopaminergic neurons is accompanied by Lewy bodies (Tompkins et al., 1997; Burke and O’Malley, 2013). Patients with mutations in α-SYN present [Parkinson disease (PARK)1, PARK3/4/5] or not present (PARK2 and PARK8) Lewy bodies associated with nigral degeneration (Foltynie et al., 2002b; Duce et al., 2017). Mutations such as PARK1 lead to amino acid changes such as A53T that increase α-SYN aggregation to form oligomers and fibrils (Duce et al., 2017). DA inhibits the transition of the protein oligomers neurotoxic to filaments, a property that may clarify the higher vulnerability of dopaminergic cells to neurodegeneration in PD (Foltynie et al., 2002b). Moreover, neurons in the SN, regardless of whether they contain Lewy bodies or not, present morphological dendritic abnormalities or biochemical changes, indicating that all neurons are involved in the disease process (Patt et al., 1991; Bergeron et al., 1996; Hill, 1996; Devi et al., 2008). Due to its structure, α-SYN can interact with anionic lipids, which leads to conformational changes that facilitate its aggregation into toxic species (Schapira and Jenner, 2011; Bose and Beal, 2016; Shamoto-Nagai et al., 2018; Zeng et al., 2018; Gilmozzi et al., 2020). For instance, the accumulation of mutant forms of α-SYN in the inner mitochondrial membrane disrupts complex I, increasing the production of reactive oxygen species (ROS) and contributing to neuronal apoptosis (Devi et al., 2008). ROS influence cellular self-defenses by promoting the cytoprotective effects of DJ-1 and PTEN-induced putative kinase 1 (PINK1) while inducing Akt dysregulation (Zhao et al., 2017).

## Why Are Dopaminergic Neurons in the Midbrain So Vulnerable?

Dopaminergic neurons in the midbrain have unique morphological characteristics that may contribute to their enhanced vulnerability (Carlsson and Fornstedt, 1991; Chung et al., 2005; Alavian et al., 2008; Hegarty et al., 2013). For example, DA neurons have long unmyelinated axons and massive dendrites that branch out into SNpr, with their somas being less than 1% of the total volume of these cells (Iarkov et al., 2020). Due to this morphology, a relatively small number of neurons provide massive dopaminergic innervation of the striatum (Sulzer, 2007). It has been calculated that each neuron in the SN may have up to 150,000 presynaptic terminals in the striatum (Oorschot, 1996; Sulzer and Schmitz, 2007). The normal functioning of such neurons requires highly active axonal transport through microtubules to support metabolic and reparative processes, synaptogenesis, removal of cellular waste, and communication with other brain cells (Prots et al., 2013, 2018; Lu et al., 2014). These cellular process demands high levels of ATP, turning DA neurons in the SN exceptionally susceptible to mitochondrial dysfunction during the development of PD (Horowitz et al., 2011; Venkateshappa et al., 2012; Vanhauwaert and Verstreken, 2015; Course and Wang, 2016; Burbulla et al., 2017).

## Molecular Mechanisms Associated With PD

It is reasonable to postulate that an accumulation of risk factors above the repair capacity of DA neurons triggers mitochondrial dysfunction, abnormal accumulation of misfolded proteins, oxidative stress, and tau hyperphosphorylation in the PD brain (Alexander, 2004; Perier and Vila, 2012; Franco-Iborra et al., 2016; Jiang and Dickson, 2018). Tau dysfunction disrupts the potential of the mitochondrial membrane, impairs the activity of respiratory enzymes, resulting in a decreased ATP production and energy supply as well as increased reactive oxygen species (ROS) production (O^2–^and H_2_O_2_) (Bose et al., 2011; Keane et al., 2011; Sutachan et al., 2012). Oxidative stress damages cellular organelles and the DNA, an event that is particularly dangerous for mitochondrial DNA that does not have protective histones and therefore is more vulnerable to ROS damage than nuclear DNA (Dexter and Jenner, 2013). Once started, the disease develops on the principle of positive feedback; oxidative stress can potentiate different risk factors, such as age and unfavorable environmental conditions to induce mutations in both cellular and mitochondrial DNA (Bandy and Davison, 1990). Although mitochondria contain the genetic information to produce proteins, most mitochondrial proteins, including those involved in DNA transcription, translation, and repair, are encoded by nuclear DNA and transported to mitochondria from the cytosol (Lenka et al., 1998; Lee et al., 2005). DNA mutations affecting genes involved in mitochondrial electron transport, glucose utilization, and glucose sensing may correlate with PD occurrence (Blanch et al., 2016; Requejo-Aguilar and Bolanos, 2016; Grunewald et al., 2019). It has been found that 28 sets of genes are linked to PD, likely playing a pathogenic role at the early stages of the disease (Anderson and Becker, 1981; Zheng et al., 2010; Keane et al., 2011). Currently, a more extensive list of genes is associated with the onset of PD, supporting the multifactorial etiology of both familial and sporadic cases of PD (Scott et al., 2017; Lu et al., 2018; Zeng et al., 2018; Kline et al., 2020; Wang et al., 2020b; Allende et al., 2021; Martinez-Banaclocha, 2021).

An early study investigating changes in the binding of the α4β2 nAChR tracer 5- (125)I-A-85380 in PD found a loss of striatal 5-(125)I-A-85380 binding that correlated with the loss of nigrostriatal dopaminergic markers (Pimlott et al., 2004). Similar changes were observed in subjects with dementia with Lewy bodies (DLB) that showed a reduced striatal 5-(125)I-A-85380 binding density, which the authors interpreted as an early degeneration in nigrostriatal inputs. These results suggest the involvement of the nAChRs on PD etiology (Pimlott et al., 2004). In agreement with this idea, multiple epidemiological studies have shown that active smokers have a lower risk of developing PD (Fratiglioni and Wang, 2000; Quik, 2004; Chapman, 2009; Chen et al., 2010; Greenbaum et al., 2013; Gallo et al., 2019; Cheng and Wang, 2020; Kim et al., 2020; Mappin-Kasirer et al., 2020) encouraging the investigation of the potential neuroprotective effects of alkaloids such as nicotine and other nicotinoids from tobacco plants with positive results (Maggio et al., 1998; Linert et al., 1999; Court et al., 2000; Mihailescu and Drucker-Colin, 2000; Quik and Kulak, 2002; Soto-Otero et al., 2002; Quik et al., 2006; Park et al., 2007; Bordia et al., 2008; Huang et al., 2009). This effect has been attributed mainly to nicotine or its metabolites acting on the AChRs (Bordia et al., 2008; Huang et al., 2009). On the other hand, nAChRs are expressed on every cell of the dopaminergic system and exert many neuroprotective effects. For this reason, modulators of the nAChRs may act as preventative drugs against PD deserve more in-depth consideration (Parain et al., 2001, 2003; Soto-Otero et al., 2002; Bordia et al., 2008; O’Leary et al., 2008; Riveles et al., 2008; Hong et al., 2009; Huang et al., 2009, 2011b; Bordia et al., 2010; Quik et al., 2012, 2013a; Barreto et al., 2014; Iarkov et al., 2020).

On the other hand, other authors have attributed these potential positive effects of tobacco consumption in decreasing the risk for PD to the content of lithium in the cigarettes (Guttuso, 2019; Guttuso et al., 2019a, b). These effects have been linked to changes in the activity of beta-Catenin, a transcriptional cofactor that upregulates the expression of canonical Wnt target genes, that it has been found reduced in sporadic PD and cell carrying Leucine-rich repeat serine/threonine-protein kinase (LRPK)2 and beta-glucosidase PD-linked mutations (Marchetti, 2018). Also, smokers’ brains have significantly lower alpha-synuclein levels. Tobacco contains very high lithium levels compared to other plants. Lithium has a broad array of neuroprotective actions, including enhancing autophagy and reducing intracellular alpha-synuclein levels, and is effective in neurotoxin and transgenic preclinical PD models (Guttuso, 2019; Guttuso et al., 2019a, b; Vallee et al., 2021). One of the lithium’s neuroprotective actions is the enhancement of beta-catenin-mediated activity, leading to increased Nurr1 expression through its ability to inhibit glycogen synthase kinase-3 beta (GSK3β) (Zhu et al., 2014; Guttuso, 2019; Guttuso et al., 2019b; Vallee et al., 2021). The authors hypothesized that inhaled lithium from smoking might account for the associated reduced rates of PD, a beneficial effect mediated by the inhibition of GSK3β and activation of beta-catenin, two factors that could be effective therapeutic targets against PD, for neuroprotective drugs, including the ones modulating the α7nAChRs (L’Episcopo et al., 2014; Liu et al., 2017; Guttuso, 2019; Guttuso et al., 2019b; Vallee et al., 2021).

## Neurotransmitter Systems in the Striatum Altered by PD

The striatum receives many synaptic inputs from all cortical regions and the thalamus providing excitatory glutamatergic afferents (Aosaki et al., 2010; Ferre et al., 2010; Huang et al., 2011a, b). At the same time, the nigrostriatal pathway delivers modulatory neurotransmitters such as DA, ACh, GABA, nitric oxide, and adenosine (Calabresi et al., 2000a; Morelli et al., 2010; Parent et al., 2011; Tripathy et al., 2015; Sanjari Moghaddam et al., 2017; Lopes et al., 2019). All these neurotransmitter systems modulate the efficacy of the synaptic transmission in the striatum, which processes excitatory glutamatergic signals from cortical and thalamic afferents and modulates signals from dopaminergic neurons of the midbrain, aspiny GABAergic, and cholinergic interneurons (Bolam et al., 2000; Kreitzer and Malenka, 2008; Gerfen and Surmeier, 2011). These signals are received and processed by the dorsal striatum MSN, which make up 90–95% of the striatum neuron population (Tepper et al., 2007). The remaining 5–10% of striatum neurons are interneurons, including the GABA and ACh interneuron (ChI) populations, which are significant regulators of both MSN and striatal afferents (Durieux et al., 2011; Munoz-Manchado et al., 2018). Among them, the most important are ChIs, which closely interact with DA afferents of the midbrain (Kim et al., 2019; Dautan et al., 2020). The glutamatergic, serotonergic, cholinergic, GABAergic, noradrenergic systems are involved in modulating the striatum’s output signals (Calabresi et al., 2000a; Do et al., 2012; Zhai et al., 2019). In addition, opioids, neuropeptides, steroids, and adenosine receptors families are present in the dorsal striatum (Aosaki et al., 2010; Ferre et al., 2010; Huang et al., 2011b; Moreno et al., 2011; Quik et al., 2012, 2013a; Almey et al., 2015; Iarkov et al., 2020). Due to the presence of such a variety of modulators, DA deficiency could be surmounted by modulating these receptors (Quik and McIntosh, 2006; Quik et al., 2007; Livingstone and Wonnacott, 2009; Avena and Rada, 2012; Goldberg et al., 2012; Mathur and Lovinger, 2012; Myslivecek et al., 2017; Ferre and Ciruela, 2019; Ztaou and Amalric, 2019; Liu, 2020).

## Interaction of the Cholinergic and Dopaminergic Systems in the Striatum

**FIGURE 1:**
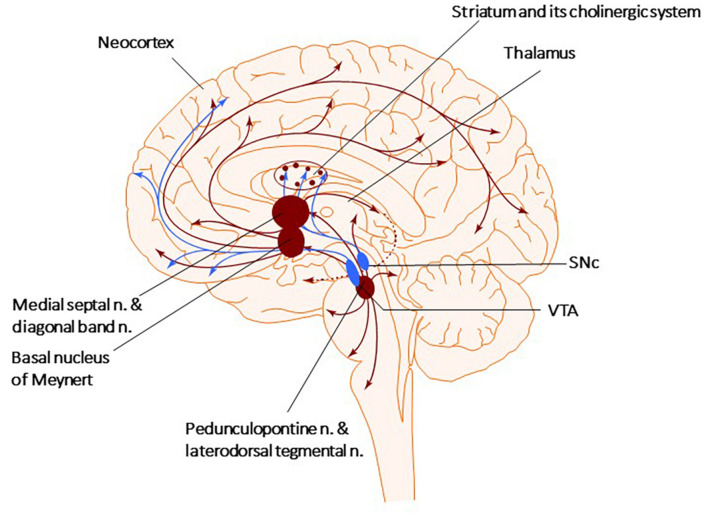
Cholinergic and Dopaminergic systems. The diagram describes the Cholinergic (brown) and Dopaminergic (blue) systems. There are four primary sources of cholinergic projections in the mammalian brain. These include pedunculopontine nucleus and laterodorsal tegmental nuclei; a set of thalamic nuclei; striatum, where few cholinergic neurons are local interneurons; and the basal forebrain nuclei, which collectively serve as the primary sources of cholinergic projection neurons in the neocortex, hippocampus, and amygdala. SNc-Substantia nigra pars compacta, VTA, ventral tegmental area.

Both the dopaminergic and cholinergic systems belong to the regulatory systems of the brain, the neurons of which are actively involved in maintaining the body’s homeostasis (Picciotto et al., 2012; Rizzi and Tan, 2017). They have a similar anatomical structure with the neuronal bodies of both systems located in the brain stem, midbrain, and subcortical structures of the forebrain, and they send their axons throughout the forebrain toward the cortex hippocampus, and limbic structures (see Figure 1). Both express several different types of receptors that can generate a wide range of cellular responses (Rizzi and Tan, 2017). It is essential to keep in mind that during the development of PD, the brain loses not only dopaminergic neurons but also cholinergic and serotonergic neurons (Reader and Dewar, 1999; Roselli et al., 2010; Ferrer et al., 2012; Myslivecek, 2021).

There are four primary sources of cholinergic projections in the mammalian brain: the pedunculopontine (PPN) and laterodorsal tegmental nuclei, the thalamic nuclei, the striatum, where a small number of giant cholinergic neurons play the role of local interneurons, and the basal forebrain nuclei, that collectively serve as crucial sources of cholinergic neurons projecting toward the neocortex, hippocampus, and amygdala (Figure 1; Garcia-Rill, 1986; Charara and Parent, 1994; Bohnen et al., 2011; Stein and Aziz, 2012; Dautan et al., 2014). The PPN can be divided into two subnuclei, the pars compacta (PPNc) and pars dissipatus (PPNd), and it is involved in starting and modulating stereotyped movements, including gait (Garcia-Rill, 1986; Snijders et al., 2016; Dos Santos et al., 2021). Glutamatergic neurons of the PPNd (pars dissipatus) regulates the basal ganglia and spinal cord. In contrast, the cholinergic pars compacta (PPNc) is part of the loop connecting the spinal cord and limbic areas with the basal ganglia and thalamus (Bohnen et al., 2011; French and Muthusamy, 2018; Bertino et al., 2020). Non-bursting cholinergic PPNc neurons are considered key to sustaining steady-state locomotion (Brimblecombe et al., 2018; Sharma et al., 2020; Huerta-Ocampo et al., 2021). Additionally, small cholinergic neurons are present in the reticular formation, the medial habenula, and the cortex (Mesulam et al., 1983, 1992; Terenzi et al., 1992; Ballinger et al., 2016).

The cholinergic neurons in the striatum play one of the most critical roles in developing symptoms in PD, and their stimulation decrease PD symptomatology (Bohnen et al., 2009; Dautan et al., 2014; Kucinski and Sarter, 2015; Osada and Iwasaki, 2017; Chambers et al., 2019; Lieberman et al., 2019). As mentioned above, the striatum contains giant aspiny ChIs, connecting to medium spiny neurons. Although giants ChIs account for only 1–3% of striatal neurons, they have highly dense axonal arbors that overlap with those of dopaminergic neurons projecting from the SNpc (Dautan et al., 2014, 2020; Mallet et al., 2019; Martel and Apicella, 2021). Thus, the high density of striatal cholinergic markers reveals the vital role of the cholinergic neurotransmission in modulating striatal function (Phelps et al., 1985; Calabresi et al., 2000a, b; Mallet et al., 2019; Martel and Apicella, 2021).

Research over the past decade shows that striatal ChIs maintain synaptic plasticity and are involved in memory and other cognitive functions mediated by the posterior striatum, such as attention and motivation (Bennett et al., 2000; Bohnen et al., 2011; Havekes et al., 2011; Deffains and Bergman, 2015). ChIs display a constant spiking activity in the absence of synaptic inputs (Bennett et al., 2000; Goldberg and Reynolds, 2011). Changes in ChI activity occur during associative conditioned learning (Robinson et al., 2011; Jiang et al., 2016). For example, in classical conditioning studies, the temporal pattern of ChIs activity has been investigated. In these experiments, animals learned to associate a conditioned stimulus (CS) (a tone) with an unconditioned stimulus (US) (a food reward) (Wilson and Fadel, 2017; Kellis et al., 2020). The results showed that during conditioning, shortly after the presentation of the CS, ChIs typically responded with a pause in firing that lasted approximately 200 ms. This pause was preceded or followed by an excitatory burst response (Mallet et al., 2019). This evidence supports the view that ChIs are involved in acquiring contextual information during conditioning learning (Aosaki et al., 1994a, b; Apicella, 2017). ChIs modulation with a cholinergic agonist mimicked the electrical oscillations in the striatum of PD brains (McCarthy et al., 2011). Furthermore, Opto-excitation of ChIs in healthy animals resulted in PD-like motor deficits (Kondabolu et al., 2016), while ChIs Opto-inhibition decreased them in PD mice (Maurice et al., 2015). Other scientists have investigated how the temporality of ChIs activity shapes striatal function using optogenetics coupled to the direct infusion of cholinergic modulators in the striatum (Mallet et al., 2019). Overall, this evidence suggests that both ChI and dopaminergic neurons work together to regulate some motor and cognitive functions and represent promising targets for alleviating the symptoms in PD (Iarkov et al., 2020).

## The Development of an Imbalance Between Different Systems in the Striatum as the Main Contributing Factor in PD

Dopamine deficiency in the striatum causes an imbalance of activity between two MSN populations, each expressing only one type of receptor (D1R or D2R). Each of both MSN groups has a unique path to the GPi/SNpr neurons. MSNs expressing D1R form a direct pathway, while those expressing D2R form an indirect pathway via the GPe and the subthalamic nucleus (STN) (Alexander, 2004; Tozzi et al., 2011; Lu et al., 2021). DA deficiency causes a decrease in the activity of MSN expressing D1R and increases the activity of neurons expressing D2R, thereby causing motor and cognitive dysfunctions (Alexander, 2004; Tozzi et al., 2011; Wang et al., 2019). The balance between the dopaminergic and cholinergic systems is vital for the correct functioning of the striatum (Aosaki et al., 2010; Lester et al., 2010; Crans and Ciruela, 2021). PD symptoms such as tremor and rigidity are ameliorated by L-DOPA and anticholinergic drugs, suggesting that PD is a hypercholinergic disorder induced by a dysbalance between Dopaminergic and cholinergic systems (Bohnen and Albin, 2011; Tata et al., 2014; McKinley et al., 2019).

Parkinson’s disease develops as an imbalance between dopaminergic inputs and cholinergic interneurons as well as between the serotoninergic and histaminergic systems, increasing the histaminergic tone and decreasing the serotoninergic and dopaminergic activities (Fahn, 1989; Przuntek and Muller, 1999; Aquino-Miranda et al., 2012; Johnston et al., 2019). This hypothesis is coherent because PD symptoms can be successfully relieved by anticholinergics and anti-histamine drugs such as Benadryl (Barbeau, 1962). However, the prescription of anticholinergic drugs stopped due to their undesired side effects, including the impairment of cognitive abilities (Cooper et al., 1992; Herzallah et al., 2010; Crispo et al., 2016). These results suggest that a deterioration of the ascending cholinergic neurons observed post-mortem in PD brains might underly the behavioral deficits in tasks depending on the subcortical frontal cortex. After the work of Alexander and DeLong (Alexander et al., 1986; DeLong, 2000), the concept changed, but now many researchers are again paying attention to it, and, considering that an imbalance between the ChIs activity and DA input signals contributes to the development of PD (Threlfell et al., 2012; Ztaou and Amalric, 2019). Fortunately, over the last decade, results obtained with new research methods have clarified the main aspects of the complex relationship between these two systems, clarifying that ChIs is modulated mainly by dopaminergic neurons located in the SN and the VTA (Threlfell and Cragg, 2011; Gonzales and Smith, 2015).

Direct pathway MSNs are activated by dopaminergic signals via D1R and inhibited by ChIs signals via M4 mAChRs in these cells expressing D1R (Bonsi et al., 2011; Gerfen and Surmeier, 2011; Oldenburg and Ding, 2011). The MSNs forming the indirect pathway are inhibited by inputs from dopaminergic neurons through D2R but activated by inputs coming from ChIs stimulating the M1 mAChRs (expressed in both MSNs expressing D1R and D2R) (Bonsi et al., 2011; Gerfen and Surmeier, 2011; Oldenburg and Ding, 2011; Goldberg et al., 2012; Rizzi and Tan, 2017). Thus, the dopaminergic system of the midbrain and striatal ChIs modulate each other to maintain the functional balance between the direct and indirect pathways, precisely controlling the movement (Liu, 2020). At the same time, dopaminergic control of ACh release depends on dopaminergic neurons on ChIs expressing D2Rs that decrease ACh release (Stoof et al., 1992; Consolo et al., 1993; Yan et al., 1997; Pisani et al., 2000). Only a tiny fraction of ChI expresses D1Rs, which increases ACh release (Damsma et al., 1991; Di Chiara et al., 1994; Steinberg et al., 1998; Acquas and Di Chiara, 1999; Lim et al., 2014; Gonzales and Smith, 2015). On the other hand, the control by the cholinergic system of DA release depends on the activation of presynaptic nAChRs and the modulation by mAChRs (Acquas and Di Chiara, 1999; Aosaki et al., 2010; Goldberg et al., 2012). Thus, the initial view of an antagonistic relationship between these two systems has evolved. New studies have shown an even higher complexity in their mutual influence that depends on an organism’s physiological state (Ztaou and Amalric, 2019; Liu, 2020). The interaction of these two systems in the striatum is perceived instead not as enmity but as a dynamic interplay in a virtual “neurotransmitter dance” (Surmeier and Graybiel, 2012).

## The Nicotinic Receptors in the Striatum

In vertebrate species, 17 different subunits of the nAChRs have been identified (α1–10, β1–4, δ, ε, γ) (Millar, 2003; Dineley et al., 2015; Papke and Lindstrom, 2020). The subunits form homo-and heteropentameric receptors, and the different combinations change their specific pharmacological properties (Wu and Lukas, 2011; Dani, 2015). The nAChRs are composed of α4, α6, α7, β2, and β3 subunits, with preferential expression of the α4β2 and α6β2 receptors (Marchi et al., 2002; Millar and Gotti, 2009; Quik and Wonnacott, 2011; Siciliano et al., 2017). Under basal conditions, binding of ligands to the α7nAChR induces a conformational change of the receptor that opens the central channel permitting the influx of sodium and calcium ions and the efflux of potassium ions (Albuquerque et al., 2009). The α7nAChRs are detectable in cortical glutamatergic terminals, potentially directly modulating corticostriatal transmission (Howe et al., 2016). In the striatum, the nAChRs are not expressed in the MSN; however, they are extensively distributed on GABAergic interneurons and dopaminergic and glutamatergic terminals, thereby having the physiological mechanism for a fine-tuned modulation of the striatum (Marshall et al., 1997; Kaiser and Wonnacott, 2000; Howe et al., 2016; Siciliano et al., 2017). Additional studies showed that activation of nAChRs, stimulated the GABAergic interneurons (English et al., 2011; Luo et al., 2013), indirectly affecting the striatal dopaminergic activity (Adermark et al., 2011; Clarke and Adermark, 2015).

## What Are the Mechanisms Involved in the Putative Neuroprotective Effects of Tobacco Consumption?

The nAChRs have attracted particular interest among researchers after numerous epidemiological studies have confirmed the low incidence of PD in active smokers (Chen et al., 2010; Gallo et al., 2019). The activation of the cholinergic system is the best target to induce neuroprotection by nicotine-derived compounds in PD (Quik et al., 2011, 2015a, b; Zhang et al., 2013; Bordia et al., 2015; Jurado-Coronel et al., 2019; Iarkov et al., 2020). ACh and other ligands acting on the AChRs stimulate the release of DA in the striatum, reduce neuroinflammation and gliosis, and promote neuronal survival and synaptic plasticity in the brain (Zhou et al., 2003; Quik et al., 2007; Goldberg et al., 2012; Jurado-Coronel et al., 2016; Abudukeyoumu et al., 2019; Bordia and Perez, 2019; Liu, 2020). Distinctively, mAChRs are expressed exclusively in the MSNs (Zhou et al., 2003; Goldberg et al., 2012).

The nAChRs are present in dopaminergic neurons, glutamatergic neurons, cholinergic interneurons (ChIs), and GABAergic interneurons in the striatum (Tanaka et al., 2010; Searles Nielsen et al., 2012; Mappin-Kasirer et al., 2020).

Since tobacco smoke contains nicotine, an agonist of the nAChRs, its protective effect has been related to the activity of nicotine on these receptors (Quik and McIntosh, 2006; Quik et al., 2007, 2009, 2012, 2013a, b; O’Leary et al., 2008; Riveles et al., 2008; Hong et al., 2009; Huang et al., 2009, 2011b; Kyaw et al., 2013; Barreto et al., 2014; Tiwari et al., 2015). In the last decades, new evidence highlights cotinine’s neuroprotective actions, a derivative of nicotine that is a positive modulator of the α7nAChRs (Soto-Otero et al., 2002; Buccafusco and Terry, 2003; Terry et al., 2005; Echeverria and Zeitlin, 2012; Barreto et al., 2014; Gao et al., 2014; Boiangiu et al., 2020; Iarkov et al., 2020). Cotinine, acting on the α7nAChRs, stimulates mechanisms of neuroprotection acting on glial cells (Morioka et al., 2018; Oliveros-Matus et al., 2020). The actual evidence suggests that activated microglia (M1 microglia) contributes to PD development (Bayarsaikhan et al., 2015; Saitgareeva et al., 2020). Thus, decreasing microglial activation could be an excellent therapeutic strategy for preventing or treating PD.

α7nAChRs are unique targets to diminish the synthesis of proinflammatory molecules and neuroinflammation due to their ability to inhibit microglial activation (Kim et al., 2015; Lee et al., 2015). Also, in microglia, the glutamate transporter (GLAST) is upregulated by α7nAChRs stimulation through the activation of both inositol triphosphate-Ca^2+/^calmodulin-dependent protein kinase II (CaMKII) and fibroblast growth factor-2 (FGF2) pathways (Morioka et al., 2014). The activation of microglial α7nAChRs is neuroprotective by inhibiting the expression of proinflammatory molecules and preventing excitotoxicity by promoting glutamate clearance (Morioka et al., 2018).

## The Neuroprotective Role of the nAChRs

The cholinergic system plays a vital role in controlling the release of neurotransmitters, decreasing neuroinflammation, promoting synaptic plasticity, and neuronal survival in the brain (Van Beek and Claassen, 2011; Hampel et al., 2018). In addition, the cholinergic system modulates both innate and adaptive immune responses (Bosmans et al., 2017; Halder and Lal, 2021; Pohanka, 2021). The cholinergic system affects immune cell proliferation, T-helper differentiation, antigen presentation, and cytokine production (Fujii et al., 2017; Halder and Lal, 2021; Lu and Wu, 2021). In agreement with these functions, the α7nAChRs are expressed in basophils, dendritic cells, macrophages, mast cells, and T and B lymphocytes (Sato et al., 1999; Skok et al., 2003; Sudheer et al., 2006; de Jonge and Ulloa, 2007; Mashimo et al., 2020). In addition, in neurons, presynaptic nAChRs modulate neurotransmitter release, while postsynaptic nAChRs increase neuronal firing rate, promoting long-term potentiation, considered a cellular mechanism of memory formation (Wayner et al., 1996; Fujii et al., 1999; Chen et al., 2006; Huang et al., 2008; Albuquerque et al., 2009; Kroker et al., 2011; Srivareerat et al., 2011; Nees, 2015; Echeverria et al., 2016b).

Epidemiological studies have shown a lower rate of development of PD in people who use tobacco products, suggesting that one or more tobacco-derived compounds may have neuroprotective effects (Fratiglioni and Wang, 2000; Parain et al., 2003; Hong et al., 2009). Various studies have shown that nicotine reduces the damage of cultured dopaminergic neurons (Riveles et al., 2008; Toulorge et al., 2011; Getachew et al., 2019). Other studies have shown that nicotine and its major metabolite, cotinine, have neuroprotective effects against 6-hydroxydopamine (6-OHDA) toxicity in cultured SH-SY5Y neuroblastoma cells expressing nAChRs (Pogocki et al., 2007; Riveles et al., 2008). Nicotine also showed neuroprotective effects in animal models of PD when administered before nigrostriatal damage occurs (Linert et al., 1999; Salminen et al., 1999; Huang et al., 2009; Quik et al., 2009, 2015a). The neuroprotective effects of other nAChR modulators have also been investigated with promising results (Pogocki et al., 2007; Tiwari et al., 2013; Jurado-Coronel et al., 2019).

## The Nicotinic Receptors and Neuronal Survival in the PD Brain

Numerous studies in this area have shown that these pathways promote neuronal survival, proliferation, and neurite growth as well as neurotransmitter release in the brain and other tissues (Eneroth et al., 1977; Fuxe et al., 1979; Aizenman et al., 1991; Sastry, 1995; Parain et al., 2001; Buccafusco and Terry, 2003; Koh et al., 2003; de Aguiar et al., 2013; Gao et al., 2014; Tiwari et al., 2015; Majdi et al., 2018).

The two main subtypes of nAChRs are the heteropentameric α4β2, α3β2, α7β2 receptors, and homopentameric α7 receptor, all of which are channels with high permeability to Ca^2+^ (Akaike and Izumi, 2018). An increase in the intracellular Ca^2+^ activates critical signaling pathways, linking input signals from the extracellular environment to a cellular response to maintain homeostasis (Gotti et al., 2009; Wang et al., 2012). Some studies have shown that activation of the nicotinic receptors can be neuroprotective by activating signaling pathways stimulated by Ca^2+^ (Kihara et al., 2001; Dajas-Bailador and Wonnacott, 2004; Rehani et al., 2008; Shimohama and Kawamata, 2018; Takahashi, 2020). For example, nicotine induces neuroprotection throughout the α7- and α4β2 receptors that, when activated, stimulate the expression of pro-survival genes that inhibit apoptosis and support synaptic function (Mudo et al., 2007; Buckingham et al., 2009; Shimohama and Kawamata, 2018; Papke and Lindstrom, 2020). For example, the increase in Ca^2+^ ion levels induced by ligands of the α7nAChRs activates the phosphatidylinositol-3-kinase (PI3K) signaling pathway (Kihara et al., 2001; Dajas-Bailador and Wonnacott, 2004; Rehani et al., 2008; Shimohama and Kawamata, 2018). For instance, downstream of the α7nAChR the protein kinase Fyn, a member of the Src family, stimulates the PI3K (Gergalova et al., 2014). PI3K phosphorylates Akt, which activates by phosphorylation the transcription factor cAMP response element-binding protein (CREB) (Leinninger et al., 2004; Wang et al., 2020a; Liu et al., 2021). This transcription, in turn, increases the expression of the cell survival factor Bcl-2 (Rehani et al., 2008). Also, Akt inhibits the pro-apoptotic factor GSK3β and, therefore, the phosphorylation of the microtubule-associated protein Tau (Rajmohan and Reddy, 2017; Sayas and Avila, 2021). These actions are relevant to preventing dementia because hyperphosphorylated Tau inhibits axonal transport and induces energy deficits in the brain leading to oxidative stress, synaptic deficits, and neuronal cell death (Perez et al., 2018; Compta and Revesz, 2021; Koziorowski et al., 2021). Coherent with this idea, the positive modulation of the α7nAChR by cotinine is neuroprotective against amyloid-β peptide (Aβ) toxicity *in vivo*, inhibiting GSK3β-mediated Tau phosphorylation, and activating the transcription factor CREB (required for long-term memory storage), improved memory abilities in mouse models of AD (Echeverria et al., 2011; Echeverria and Zeitlin, 2012; Patel et al., 2014; Grizzell and Echeverria, 2015; Grizzell et al., 2017). Other mechanisms of nAChR-mediated neuroprotection include activating the extracellular signal-regulated protein kinase/mitogen-activated protein kinase (ERK/MAPK) pathway (Grizzell and Echeverria, 2015). The α7nAChRs activate neuroprotective factors stimulated by Ca^2+^, including the protein kinase C (PKC) and the Ca^2+^/calmodulin-dependent protein kinase (CaMK), both of which activate CREB (Kawamata and Shimohama, 2011; Kawamata et al., 2011, 2012; Sutachan et al., 2012; Albert-Gasco et al., 2020).

**FIGURE 2:**
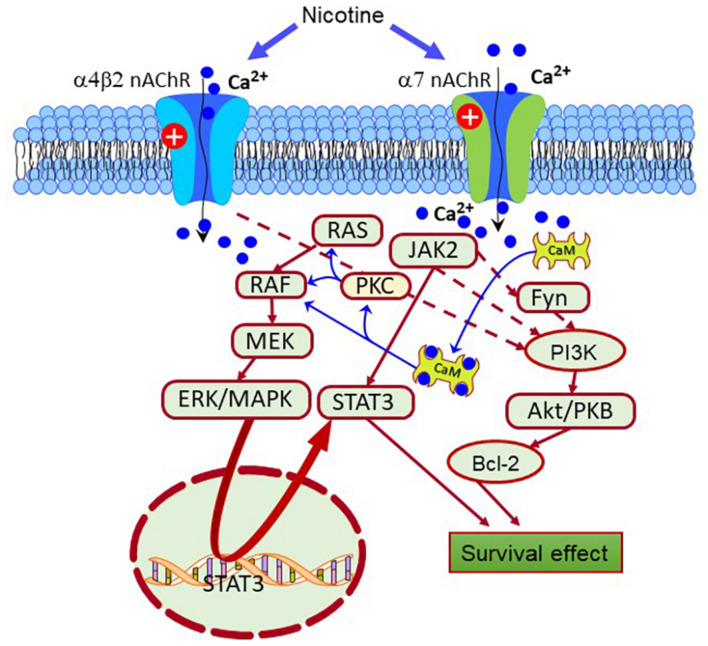
Scheme depicting different pro-survival signaling pathways activated by the nAChRs. Nicotine-induced neuroprotection is mediated by receptors, primarily through the α7 and α4β2 receptors. Also, the nAChRs can activate intracellular pathways that enhance the expression of pro-survival proteins that inhibit apoptosis. From them, Janus kinase 2 (JAK2), Fyn, protein kinase C (PKC), and calcium-calmodulin kinase (CaMK) are crucial protein factors triggering the activation of the extracellular signal-regulated protein kinase (ERK), JAK2/signal transducer, and activator of transcription 3 (STAT3), and phosphatidylinositol 3-kinase (PI3K)-Akt pathways. In turn, these pathways enhance the expression of antiapoptotic factors such as Bcl-2.

Evidence obtained using agents inducing PD-like pathologies such as Rotenone, 6-OHDA, and 1-methyl-4-phenyl-1,2,3,6-tetrahydropyridine (MPTP), enlightened potential mechanisms of nAChRs-mediated neuroprotection (Kawamata et al., 2011). These mechanisms involve activating the signal transducer and activator of transcription (STAT)1/3/5, and the Fyn/PI3K/Akt/Bcl2, Janus kinase 2 (JAK2)/PI3K/Akt (Bharadwaj et al., 2020). Besides, the stimulation of the α4β2 and α7nAChRs triggers other neuroprotective signaling cascades without the direct involvement of the PI3K system, such as the ERK/MAPK and JAK2/STAT3 pathways, PKC/Raf/MEK/ERK/STAT3, and Ras-Raf-ERK signaling pathways (Figure 2; Buckingham et al., 2009; Zdanowski et al., 2015).

The α7nAChRs also stimulate the expression and activity of various growth factors and their receptors, such as the vascular endothelial factor (VEGF) and the VEGF receptor 2 (VEGFR2), the platelet-derived growth factor (PDGF) and the PDGF receptor (PDGFR), and the epidermal growth factor receptor (EGFR) (Pillai and Chellappan, 2012). EGFR activates the Akt pathway and its downstream effectors, X-linked inhibitor of apoptosis protein-survivin and the nuclear factor kappa B (NFκB) (Zdanowski et al., 2015). In general, the nAChRs can activate neuroprotective signaling cascades in neurons, astroglia, and microglia to promote cell survival, synaptic plasticity and maintain brain homeostasis (Picciotto et al., 2000; Echeverria and Zeitlin, 2012; Kawamata et al., 2012). For example α7nAChRs upregulate the transcription factors hypoxia-inducible factor-1 (HIF-1), GATA-3, NFκB, and signal transducer and activator of transcription (STAT) 1 (Picciotto et al., 2000; Echeverria and Zeitlin, 2012; Kawamata et al., 2012; Pillai and Chellappan, 2012; Echeverria et al., 2016a, b).

## The Muscarinic Receptors in the Striatum

In the striatum, every type of neuron expresses different subtypes of both mAChR and nAChRs (Threlfell et al., 2010; Lim et al., 2014). The mAChRs are metabotropic receptors that indirectly control the activity of membrane ion channels through heterotrimeric G-proteins (Kruse et al., 2014; Roth, 2019). These G-proteins are composed of Gα and Gβg subunits classified according to the type ofα subunit, which determines their association to specific G-protein coupled receptors (GPCR) (Huang and Thathiah, 2015; Roth, 2019).

In the CNS, the mAChRs are categorized into five subtypes groups (M1 to M5) (Lim et al., 2014; Mallet et al., 2019). These receptors show significant differences in expression M1 > M2 > M4 > M3 and M5 (Zhou et al., 2003; Graef et al., 2011).

A study using atropine to inhibit M2 and M3 mAChRs present on the glutamatergic terminals revealed a small but significant increase in corticostriatal transmission, suggesting the existence of tonic cholinergic presynaptic inhibition of this excitatory afferents inputs (Pakhotin and Bracci, 2007; Mallet et al., 2019). At different, the M1 mAChR blocker pirenzepine decreased corticostriatal transmission (Tozzi et al., 2011).

In PD, anti-muscarinic receptor drugs were the first symptomatic PD treatment before discovering L-DOPA (Fahn, 1989). mAChR antagonists were used as early treatments and are still under use in PD (Langmead et al., 2008a, b; Thomas et al., 2009). The muscarinic antagonists decrease the hyperactivity of ChIs and corticostriatal glutamatergic neurotransmission after nigrostriatal denervation (Lim et al., 2014). However, while they provide some benefits, these drugs are not without side effects, including cognitive impairment (Drachman and Leavitt, 1974; Yamamoto et al., 2011). Therefore, there is a need for more selective cholinergic modulators with improved therapeutic properties. In addition, therapies with more selective modulators of the cholinergic receptors may permit more target specificity and improved pharmacokinetics compared to ACh.

## The Muscarinic AChRs Signaling and Their Role in Maintaining Brain Homeostasis

The M1, M3, and M5 mAChRs are associated with G-proteins’ Gq/11 subfamily (Sil’kis, 2003; Tobin and Budd, 2003; Santiago and Abrol, 2019). When activated by these receptors, Gq interchanges GTP for GDP and dissociates in their constituent subunits that become free to activate downstream effectors such as the phospholipase C (PLC) (Falkenburger et al., 2010). PLC activity results in the release of inositol-triphosphate 3 (IP3) and diacylglycerol (DG) (Berridge, 2016). IP3 binds to the IP3R in the endoplasmic reticulum (ER), triggering the release of Ca^2+^ from intracellular stores, and DG stimulates the PKC (Rehman and Dimri, 2020).

The activation of M2 and M4 receptors activates the Gi/o subfamily of G proteins, increasing the opening time of potassium channels and decreasing cAMP production (Santiago and Abrol, 2019). Since this second messenger activates the pro-survival PKA/CREB pathway, this decrease inhibits cell survival and synaptic plasticity in the brain (Resende and Adhikari, 2009). The M2 mAChRs also weakly bind to Gs and Gq, acting as a negative autoreceptor leading to decreased ACh release (Quirion et al., 1990; Wess et al., 2007; Santiago and Abrol, 2019).

On the other hand, the activation of the M1 mAChRs increases the non-amyloidogenic processing of the amyloid precursor protein (APP) to generate sAPPα, a proteolytic product that promotes neuroprotection by stimulating neuritogenesis, neurogenesis, synaptic plasticity, and memory formation while reducing Aβ and Tau pathology in the brain (Deng et al., 2015; Habib et al., 2017).

Some drugs, including non-steroidal anti-inflammatory compounds, have neuroprotective effects by shaping APP processing (Avramovich et al., 2002; Yogev-Falach et al., 2003; Kalaitzakis et al., 2008; Miklossy et al., 2008; Shimohama and Kawamata, 2018). Also, other kinases such as PKC and ERK1/2 stimulate the non-amyloidogenic processing of APP by α-secretase (Wang et al., 2016; Zhang et al., 2019).

**FIGURE 3:**
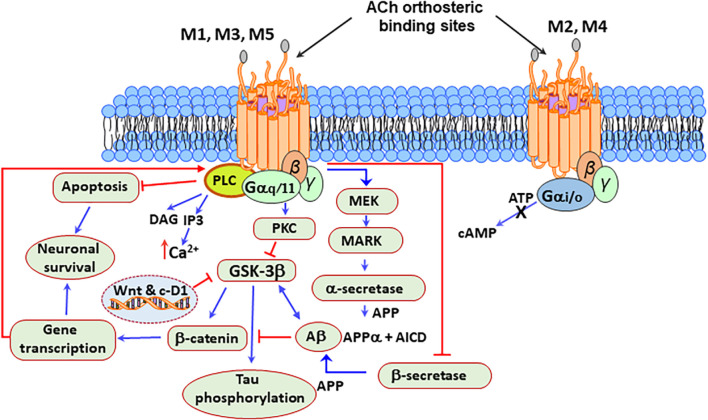
Neuroprotective signaling pathways stimulated by the mAChRs. M1, M3, and M5 mAChRs are associated with the Gq/11 subfamily of G proteins, which are responsible for the increase of cytosolic Ca^2+^, activation of phospholipase C (PLC) and protein kinase C (PKC), which leads to the production of signaling molecules inositol triphosphate (IP3) and diacylglycerol (DAG). Acetylcholine (ACh) activation of the M2 and M4 receptors, which are associated with the Gi/o subfamily of G proteins, increases the opening time of potassium channels and decreases the production of adenosine-3′,5′-cyclic monophosphate (cAMP). Besides, the stimulation of the M1 mAChRs by agonists or ACh increases the production of sAPPα and decreases the production of amyloid Aβ peptide. Protein kinase C (PKC) and the extracellular signal-regulated protein kinase (ERK)1/2 are involved in this process by activating α-secretases. The activation of the M1 mAChRs counteracts Aβ-induced neurotoxicity via the Wnt signaling pathway, as Aβ inhibits this pathway through the destabilization of β-catenin. In contrast, stimulation of M1 mAChR inactivates glycogen synthase kinase 3 (GSK3β) via PKC activation, thus stabilizing β-catenin and inducing the expression of the Wnt-targeting and cyclin-D1 genes for neuronal survival.

Also, M1 mAChR activation upregulates the expression of β-secretase 1 (BACE1) through a mechanism involving the activation of MEK/ERK, by a mechanism prevented by M2 mAChRs activation (Zuchner et al., 2004). Besides, the stimulation of M1 mAChRs counteracted the Aβ-induced inhibition of Wnt signaling by GSK3β, resulting in the stabilization of β-catenin and increased expression of survival genes (Farias et al., 2004; Jiang et al., 2014b; Wysocka et al., 2020). Additionally, the M1 mAChRs, by inhibiting the tau kinase GSK3β also prevented tau hyperphosphorylation and toxicity (Jiang et al., 2014b). Figure 3 represents a possible mAChR signaling mechanism that protects brain cells from mitochondrial dysfunction, caspase activation, oxidative stress, and DNA damage in PD.

In agreement with this effect, cell studies indicate that Wnt/β-catenin pathway inhibition mediates manganese-induced neurotoxicity (Jiang et al., 2014a). In AD, increased levels of aggregated forms of Aβ seem to interfere with the function of M1 mAChRs by uncoupling the receptor-G protein complex (Janickova et al., 2013).

**FIGURE 4:**
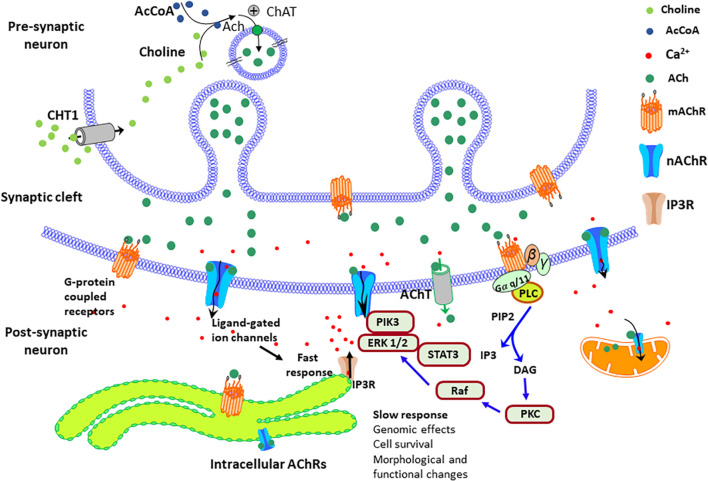
Simplified diagram of the AChRs signal transduction pathways in the pre-and post-synaptic sites. In brain cells, intracellular AChRs – both mAChRs and nAChRs are located on both the surface of the plasma membrane and intracellular membranes of various organelles, such as endosomes, Golgi apparatus, endoplasmic reticulum, mitochondria, and nuclear membranes. The choline transporter-like protein1 (CHT1) uptake choline at the presynaptic, and the acetylcholine (ACh) transporter (AChT) uptake ACh at the post-synaptic site. The entrance of calcium throughout the nAChRs stimulates signaling factors, such as the enzymes phosphatidylinositol 3-kinase (PI3K)/extracellular signal-regulated protein kinase (ERK)1/2/signal transducer and activator of transcription 3 (STAT3). These factors prevent apoptosis and stimulate the expression of prosurvival genes, The signaling cascades elicited by the mAChRs stimulate the release of calcium from intracellular stores via the activation of PLC that generates the Inositol trisphosphate (IP3) that activates the IP3 receptors (IP3Rs) and the second messenger diacylglycerol (DAG) that stimulates the protein kinase C (PKC)/Raf/ERK pathway.

On the other hand, the cellular localization of these cholinergic receptors also may play a key role in their function and cellular effects (Anisuzzaman et al., 2013; Uwada et al., 2014; Uspenska et al., 2017; Jong et al., 2018; Muramatsu et al., 2018). Although most cholinergic receptors are located in the plasma membrane to convert extracellular signals into intracellular ones, several studies have reported nAChRs in other neuronal organelles like mitochondria (Skok and Lykhmus, 2016). The current evidence suggests that intracellular α7β2 receptors mainly stimulate the PI3K/Akt pathway, while α3β2 and α4β2 receptors inhibit Akt signaling and Ca^2+^/calmodulin-dependent pathways, consequently promoting mitochondrial apoptosis (Gergalova et al., 2014; Lykhmus et al., 2014; Muramatsu et al., 2018). New studies indicate that the mAChRs, the cannabinoid receptor, and the metabotropic glutamate receptor 5 (mGluR5) also localize intracellularly in the membranes of various organelles (Jong et al., 2018). In these intracellular locations, they can transmit signals from structures such as endosomes, Golgi apparatus, endoplasmic reticulum, mitochondria, and nucleus (Boivin et al., 2008; Jong et al., 2009; Benard et al., 2012; Gergalova et al., 2014; Lykhmus et al., 2014). Recent studies have shown that approximately half of the M1 mAChRs are in the intracellular part of the membrane in neuronal cells (Anisuzzaman et al., 2013; Uwada et al., 2014). Expression studies using immunohistochemistry methods indicated that the mAChRs could also localize in the Golgi apparatus (Muramatsu et al., 2018). Interestingly, the intracellular localization of the mAChRs requires a C-terminal tryptophan motif that is only present in the M1 subtype. The M1 mAChRs are also present in postsynaptic neurons (Anisuzzaman et al., 2013; Morishima et al., 2013; Uwada et al., 2014; Muramatsu et al., 2018). Figure 4 shows a simplified schematic view of the plasma membrane and intracellular M1 mAChRs in the pre-and postsynaptic neurons, their predicted signal transduction pathways, and the elicited physiological responses. Studies with the GABAA receptor’s competitive antagonist bicuculline suggested that M3 and M4 receptors modulate DA release via facilitation or inhibition of striatal GABA release.

## Effect of Cotinine Preventing Amyloid-β Peptides Accumulation and Promoting Synaptic Plasticity in the Brain

Despite their structural similarities, nicotine, and cotinine, differ in their mechanism of action, properties, behavioral effects, and toxicity profile (nicotine is one hundred times more toxic than cotinine) (Buccafusco and Terry, 2003; Grizzell and Echeverria, 2015; Majdi et al., 2018). The last decade of research has shown that cotinine has unique pharmacokinetic and pharmacodynamic properties, acting as a very weak nAChR agonist but a positive modulator of the α7nAChRs (Moran, 2012; Grizzell et al., 2014; Sadigh-Eteghad et al., 2020). Different from nicotine, cotinine is safe and does not elicit addictive behaviors in mammals, including humans (Yim and Hee, 1995; Hatsukami et al., 1997; Vainio et al., 1998; Zevin et al., 2000; Echeverria and Zeitlin, 2012; Thomopoulos et al., 2013). Cotinine has shown to protect astrocytes from the toxic effects of chronic and acute stress *in vivo* (Alvarez-Ricartes et al., 2018; Mendoza et al., 2018; Oliveros-Matus et al., 2020) and to prevent the loss of presynaptic proteins such as synaptophysin in the PFC and hippocampus of mice subjected to chronic stress (Grizzell et al., 2014; Grizzell and Echeverria, 2015). In addition, cotinine has shown to reduce the activation of macrophages (Rehani et al., 2008) and be neuroprotective, reducing plaque deposition, tau hyperphosphorylation, and cognitive impairment while increasing the expression of the postsynaptic density protein 95 (PSD95) in transgenic AD mice overexpressing human Aβ peptides (Terry et al., 2005, 2012, 2015; Echeverria et al., 2011; Echeverria and Zeitlin, 2012; Patel et al., 2014; Grizzell et al., 2017).

In addition, there is evidence suggesting that the activation of the nicotinic receptors by cotinine modulates the stoichiometry and expression of the nAChRs (Lester et al., 2009). A study using neuronal cells and *Xenopus* oocytes expressing nAChRs (Terry et al., 2015) showed that, like nicotine, cotinine increased the expression of α4β2 receptors on the plasma membrane and induced a change in the intracellular distribution of these receptors. Furthermore, cotinine altered the assembly of α4β2 receptors to favor the assembly of (α4)_2_(β2)_3_ receptor’s stoichiometry that has higher sensitivity to the agonists than (α4)_3_(β2)_2_ stoichiometry (Srinivasan et al., 2011). Cotinine also decreased the expression of the α6β2β3 receptors (Moran, 2012). In contrast, cotinine did not change the trafficking or expression of α6β2, α4β2α5, or α3β4 receptors (Fox et al., 2015). A previous study compared ACh alone or plus cotinine on the channel activity of the α7 nAChR. The results revealed an enhanced channel activity induced by ACh plus cotinine than the receptors treated with ACh alone (Terry et al., 2015). They also found that exposure to cotinine for 2 days, at doses found in heavy smokers, induced a moderate down-regulation of α4β2 receptors expressed in *Xenopus laevis* oocyte (Terry et al., 2015). A contemporary study, using a combination of fluorescence imaging and single-molecule measurements, showed evidence that cotinine at concentrations higher than 5 μM did not increase the receptor expression on the plasma membrane, but lower concentrations of cotinine change both the assembly and trafficking of the nAChRs (Fox et al., 2015). No doubt that these changes affect their affinity for the ligands and their function in the brain. New studies are required to define these differences in more detail.

Many studies have shown that modulators of the nicotinic receptors such as nicotine and Cotinine control the release of neurotransmitters such as serotonin (O’Leary et al., 2008) and DA (Fuxe et al., 1986) in the brain (Fuxe et al., 1979), GABA and glutamate (Yan et al., 2019) affecting brain connectivity and its function.

## Predicting the Development of PD Dementia

In addition to motor deficits, PD presents with non-motor alterations, including cognitive decline, symptoms of depression, abnormal autonomic nervous system function (dysautonomia), and psychosis (Hemmerle et al., 2012; Lindqvist et al., 2012; Grover et al., 2015; Maillet et al., 2016; Samudra et al., 2016; Hussein et al., 2021). Although memory and language dysfunctions are less evident than the observed in AD, PDD is 600% higher than in the general population. One prospective 8-year study found a cumulative prevalence of dementia of 78% among PD patients (Emre et al., 2007). In another study to a 5-year prospective study in more than 400 patients with PD, the risk factors for dementia included older age, longer disease duration, later age-at-onset, and higher daily levodopa (l-dopa) dosage (Aarsland et al., 2003). PDD, like AD, shows cognitive symptoms such as a diminution of attention and executive and visuospatial abilities (Fitts et al., 2015; Ho et al., 2020; Hussein et al., 2021). PDD seems to result from a combination of AD-like, cortical Lewy-bodies and vascular pathology induced by high homocysteine level (hyperhomocysteinemia), and dysautonomia with abnormal blood pressure (BP), breathing and digestive problems, and loss of bladder control (Zoccolella et al., 2006; Byeon, 2020; Kwon et al., 2021).

In PD, it is challenging to assess the progression to dementia. Structural changes in the PD brain could predict motor and cognitive outcomes (Byeon, 2020; Chung et al., 2021; Owens-Walton et al., 2021; Shin et al., 2021). For example, previous studies have found a correlation between cortical thinning and PD dementia (PDD) (Chung et al., 2021). The methodological advances in structural and functional brain analysis could permit the prediction of PDD development in clinical settings (Oldan et al., 2021; Owens-Walton et al., 2021; Shin et al., 2021). A recent retrospective study explored whether the assessment of cortical thickness by MRI combined with other clinical symptoms using a machine learning-based model could predict the transition from mild cognitive impairment (MCI) to dementia in PD. The study involved patients diagnosed with PD and MCI and evaluated with MRI for 8 years. Features were chosen from clinical and cortical thickness variables to support vector machine models (Shin et al., 2021). From all participants, 42 patients advanced to PDD (converters), and 75 patients did not advance to PDD (non-converters). Models exhibited fair to good predictive outcomes; however, their performances increased when models included both clinical and structural variables (AUC range, 0.80–0.88). In pair-wise comparisons, models trained with both variables obtained better achievements. The authors concluded that Cortical thickness from MRI could help forecast transition from MCI to dementia in PD with improved accuracy when combined with other clinical variables (Shin et al., 2021).

Another recent study used structural and functional MRI to elucidate pathophysiological mechanisms associated with cognitive impairment and dementia in PD (Owens-Walton et al., 2021). They specifically investigated resting-state functional connectivity and morphology of the caudate nucleus, putamen, and thalamus, in PD brains. The results revealed enhanced functional connectivity of the dorsal caudate, anterior putamen, and mediodorsal thalamic subdivisions with the frontal lobe and lower functional connectivity of the dorsal caudate with posterior cortical, and cerebellar regions. Compared to cognitively unimpaired subjects, those with mild cognitive impairment (*n* = 22) demonstrated reduced functional connectivity of the mediodorsal thalamus with the paracingulate cortex while also demonstrating increased functional connectivity of the mediodorsal thalamus with the posterior cingulate cortex, compared to subjects with dementia (*n* = 17). The patients with PDD showed a significant reduction in volume in those regions compared to controls or PD participants without dementia. The authors concluded that abnormalities in the functional connectivity of the basal ganglia-thalamocortical circuits, mainly between the mediodorsal thalamus with the cingulate regions, are involved in the appearance of dementia in PD (Owens-Walton et al., 2021). Changes in connectivity can predict the severity of the disease, with the pattern of connectivity considered a form to predict the response to L-DOPA and cognitive status in the PD patients (Amboni et al., 2015; Akram et al., 2017; Anderkova et al., 2017; Chung et al., 2021).

## Conclusion

The cholinergic system has a powerful influence over the striatum function by controlling the dopaminergic activity and promoting the survival of neurons to mitochondrial dysfunction, oxidative stress, and neuroinflammation. The dopamine-centric view of PD has failed to control the pathology, and new and more integral approaches considering the disbalance of other neurotransmitters such as serotonin, histamine, and ACh need to be addressed to prevent the loss of dopaminergic neurons in PD. Cotinine positively modulates the nAChRs, and affects the release of serotonin, DA, GABA and glutamate receptors in the brain facilitating brain connectivity and function (Stone, 2021). Based on this evidence, it is reasonable to postulate that cotinine could be a critical factor delaying cognitive impairment in PD or PDD in tobacco users. Further preclinical and clinical studies are required to fully unmask the potential beneficial effects of cotinine in PD.

## Author Contributions

All authors listed have made a substantial, direct and intellectual contribution to the work, and approved it for publication.

## Conflict of Interest

The authors declare that the research was conducted in the absence of any commercial or financial relationships that could be construed as a potential conflict of interest.

## Publisher’s Note

All claims expressed in this article are solely those of the authors and do not necessarily represent those of their affiliated organizations, or those of the publisher, the editors and the reviewers. Any product that may be evaluated in this article, or claim that may be made by its manufacturer, is not guaranteed or endorsed by the publisher.

## Abbreviations

MPTP: 1-methyl-4-phenyl-1,2,3,6-tetrahydropyridine
6-OHDA: 6-hydroxydopamine
ATP: adenosine 5′-triphosphate
α-SYN: Alpha synuclein
Aβ: Amyloid beta peptide
APP: Aβ precursor protein
AChEI: acetylcholinesterase inhibitors
AD: Alzheimer’s disease
BDNF: brain-derived neurotrophic factor
CREB: cAMP response element-binding protein
ChI: cholinergic interneurons
COX-2: cyclooxygenase 2
DA: dopamine
D1R: dopamine receptor 1
D2R: dopamine receptor
EGFR: epidermal growth factor receptor
ERK: extracellular signal-regulated protein kinase
GFAP: glial fibrillar acidic protein
GSK3: βglycogen synthase kinase 3β
GPi: Globus pallidus internal
GST: glutathione S-transferase activity
HIF-1: hypoxia-inducible factor-1
IL: Interleukin
JAK2: Janus kinase 2
MRI: Magnetic resonance imaging
MCI: Mild cognitive impairment
MAOIs: monoamine oxidase inhibitors
NF κ B: neurotrophic factor kappa B
nAChRs: nicotinic acetylcholine receptors
mAChRs: muscarinic acetylcholine receptors
NOS: nitric oxide synthase
NMDA: N-methyl -D-aspartate
PAM: positive allosteric modulator
PD: Parkinson’s disease
PARK2: Parkinson disease-2
PARK8: Parkinson disease-8
PDD: Parkinson’s disease dementia
PDGF: platelet-derived growth factor
PDGFR: PDGF receptor
PI3K: phosphatidylinositol 3-kinase
Akt: Protein kinase B
PPN: pedunculopontine
PPNd: PPN pars dissipatus
PPNpc: PPN pars compacta
PSD95: postsynaptic density protein 95
STAT: signal transducer and activator of transcription
SN: substantia nigra
SNpc: SN pars compacta
STN: subthalamic nucleus
TNF: tumor necrosis factor
VEGF: vascular endothelial growth factor
VEGFR: vascular endothelial growth factor receptor
VTA: ventral tegmental area.
